# Supplementary material for: Membrane fluidity control by the Magnaporthe oryzae acyl-CoA binding protein sets the thermal range for host rice cell colonization
Source: PLoS Pathog. 2024 Nov 25;20(11):e1012738. doi: 10.1371/journal.ppat.1012738 (PMC11627410; doi:10.1371/journal.ppat.1012738)
Supplement: S2 Table — (DOCX) [file ppat.1012738.s010.docx]

### S2 Table. Strains used in this study.

| **Strains** | **Genotype** | **Reference** |
| --- | --- | --- |
| Guy11 | *M. oryzae* wild type isolate (WT) used throughout this study | [1] |
| Δ*acb1* | WT parental strain carrying a deletion of the *ACB1* gene (MGG_06177) encoding the acyl-CoA binding protein | *This study* |
| Δ*acb1*  *ACB1* | Complementation strain resulting from integration of the full length *ACB1* gene, under native promoter, into the genome of a Δ*acb1* deletant strain | *This study* |
| Δ*acb1*  *pRP27::ACB1* | Complementation strain resulting from integration of the full length *ACB1* gene, expressed under the constitutive *RP27* promoter, into the genome of a Δ*acb1* deletant strain | *This study* |
| *PWL2-mCherry:NLS*  *BAS4-GFP* | WT strain carrying pBV591^2^ encoding Pwl2 fused to mCherry:NLS under its native promoter and Bas4 fused to GFP under its native promoter | [3] |
| Δ*acb1*  *PWL2-mCherry:NLS*  *BAS4-GFP* | Δ*acb1* strain carrying pBV591^2^ encoding Pwl2 fused to mCherry:NLS under its native promoter and Bas4 fused to GFP under its native promoter | *This study* |
| Δ*ndk1* | WT parental strain carrying a deletion of the *NDK1* gene (MGG_08622) encoding a nucleotide diphosphate kinase | [4] |

1. R. A. Wilson, N. J. Talbot, Under pressure: investigating the biology of plant infection by *Magnaporthe oryzae*. *Nat. Rev. Microbiol*. **7,** 185-95 (2009).

2. C. H. Khang et al, Translocation of *Magnaporthe oryzae* effectors into rice cells and their subsequent cell-to-cell movement. *Plant Cell* **22,** 1388-1403 (2010).

3. G. Li, N. Dulal, Z. Gong, R. A. Wilson, Unconventional secretion of *Magnaporthe oryzae* effectors in rice cells is regulated by tRNA modification and codon usage control. *Nat. Microbiol.* **8**,1706-1716 (2023).

4. R. O. Rocha, R. A. Wilson, *Magnaporthe oryzae* nucleoside diphosphate kinase is required for metabolic homeostasis and redox-mediated host innate immunity suppression. *Mol. Microbiol.* **114**: 789-807 (2020).
