# Supplementary material for: Membrane fluidity control by the Magnaporthe oryzae acyl-CoA binding protein sets the thermal range for host rice cell colonization
Source: PLoS Pathog. 2024 Nov 25;20(11):e1012738. doi: 10.1371/journal.ppat.1012738 (PMC11627410; doi:10.1371/journal.ppat.1012738)
Supplement: S3 Table — (DOCX) [file ppat.1012738.s011.docx]

**S3 Table. Oligonucleotides used in this study.**

| **Primer Name** | **Sequence** |
| --- | --- |
| M13F:IL | CGCCAGGGGTTTTCCCAGTCACGACGTCGACGTGCCAACGCCACAG |
| ILSplit | CCAAGCATGTGCAGTGCCTTC |
| LV1Split | GGAGGCCGACGTCATAGGCATC |
| M13R:LV1 | AGCGGATAACAATTTCACACAGGAGTCGACGTGAGAGCATGCTAA |
| ACBP1-1 | GAAGTTGGGCGAGGGACTG |
| ACBP1-2 | GTCGTGACTGGGAAAACCCTGGCGGGACGATAGTGTGTGCGAGAG |
| ACBP1-3 | TCCTGTGTGAAATTGTTATCCGCTGCTCAAGAGAAGTACGTTGCGCTC |
| ACBP1-4 | GATGAAATTCGGCGCTCGTTAG |
| ACBP1-nesF | GCTACGGCTCGGGCG |
| ACBP1-nesR | GGTTCCATACACACCAAAATACAGTCG |
| ACBP1-compF | TATAGGGCGAATTGGGTACTCAAATTGGTTGAAGTTGGGCGAGGGACTG |
| ACBP1-compR | CCCGGTGAACAGCTCCTCGCCCTTGCTCACGATGAAATTCGGCGCTCGTTAG |
| Rp27:ACBP1-compF | TTTCGTAGGAACCCAATCTTCAAACAAACATGGCTCCAGCACAGTC |
| Rp27:ACBP1-compR | CCCGGTGAACAGCTCCTCGCCCTTGCTCACCAACGGCCTCAGGGACCTTG |
| qPCR-*ACT1*-F | CGCTTCCGTGCTCCTGAGGCTC |
| qPCR-*ACT1*-R | CCACCGATCCAGACGGAGTACTTG |
| qPCR-*ACB1*-F | ATGTTCGACCTCAAGGGCAAGTC |
| qPCR-*ACB1*-R | TCAGGGACCTTGGCTTCGTCA |
