# Supplementary material for: Membrane fluidity control by the Magnaporthe oryzae acyl-CoA binding protein sets the thermal range for host rice cell colonization
Source: PLoS Pathog. 2024 Nov 25;20(11):e1012738. doi: 10.1371/journal.ppat.1012738 (PMC11627410; doi:10.1371/journal.ppat.1012738)
Supplement: Attachment — Submitted filename: Response to Reviewers_comments.docx [file ppat.1012738.s013.docx]

**Response to Reviewer’s comments**

We thank the reviewers for their thoughtful and insightful comments. We have addressed them through additional experimentation and clarified some areas of confusion. The main additions we have made include repeating all FAME analyses by incorporating the complementation strain; including data from an overexpressing *ACB1* strain; including the complementation strain in the FM4-64 analysis and providing quantification of the differences in FM4-64 distribution in the different strains; quantification of leaf infection severity throughout; better representing the membrane fluidity data (which no longer incorporates Amphotericin B treatments) and providing more evidence for the role of *ACB1* in membrane integrity by incorporating data from an ion leakage assay; and providing more controls for the membrane fluidity assay. We hope that these are efforts are sufficient to make this work suitable for publication in *PLOS Pathogens*.

**Part I - Summary**

*Please use this section to discuss strengths/weaknesses of study, novelty/significance, general execution and scholarship.*

*Reviewer #1: In this study the authors Richter et al., have investigated what molecular and cellular processes are required to determine the temperature range for successful biotrophic growth of the rice blast fungus Magnaporthe oryzae. This study is considerably timely considering the imposing threat climate change is having on the environment, and how these changes could effect the spread of destructive plant pathogens globally. In order to understand how the fungus may adapt to changes in temperature, the authors characterised a gene encoding an acyl-CoA binding protein ACB1, an intracellular transporter of long chain acyl-CoA esters. The mutant displayed aberrant fatty acid desaturation levels compared to wild type, and loss of pathogenicity at low (22°C) and optimal (26°C) temperatures, but this phenotype was remediated at elevated (29°C). Further analysis revealed the mutant was reduced in vegetative growth and impaired membrane trafficking at 22°C and 26°C, but not at 29°C. During biotrophic growth the mutant displayed a multi-BIC phenotype and inhibited growth at 26°C, which was remediated at 29°C. Finally, the authors showed the ∆acb1 mutant had impaired membrane fluidity at 22°C and 26°C but not elevated temperatures, suggesting ACB1 suppresses membrane rigidity at optimal and suboptimal but not elevated temperatures. In summary, the authors suggest ACB1 helps to keep the cell membrane flexible and less rigid at 22°C and 26°C, which is important for cell function. However, at higher temperatures, ACB1 is dispensable for this function. I find this study very interesting as it is suggesting rice blast disease can survive in a wider range of temperatures than previously thought. However, I do have some points that should be addressed before publication can be considered.*

We thank the reviewer for their fine comments, and we are very pleased that the work was found to be so interesting.

*Reviewer #2: This manuscript functional characterized the ACB1 gene encoding the single acyl-CoA-binding protein, an intracellular transporter of long-chain acyl-CoA esters, in Magnaporthe oryzae. The manuscript is very well-written and the data is presented in a clear and concise fashion. The introduction and discussion are rich in fundamental background information. The experimental designs, results, and conclusions are very consistent and the outcomes should serve as excellent references for fungal geneticists and plant pathologists. Thank you for putting together this fantastic research data that leads to a high-quality manuscript.*

*I strongly support the publication of this manuscript in the journal Plos Pathogens.*

We thank to reviewer for their kind and thoughtful assessment of the work.

*Reviewer #3: Strengths*

*This is a well-written, novel, and thought-provoking study that addresses the importance of an acyl-coA binding protein in influencing the thermal constraints of vegetative growth and biotrophic infection of rice by the blast fungus Magnaporthe oryzae. The authors describe the phenotypic characterization of a previously described acb1 knockout mutant with altered fatty acid membrane composition, that is perturbed in vegetative growth and virulence at normally optimal temperatures (22-26 degrees C). The authors demonstrate convincingly using live-cell imaging that acb1 KO mutants produce atypical BIC's, which are sites of cytoplasmic effector secretion, at these low, normally optimal temperatures. The authors also provide (less robust) data to support the idea that membrane trafficking is perturbed at lower, but not higher, temperatures in acb1 KO mutants. Interestingly, the authors go on to convincingly demonstrate that acb1 KO mutants can actually cause disease at high temperatures (but not low) when shifted to these higher temperatures after appressorium-mediated cuticle penetration. The authors go on to provide data from a membrane fluidity assay, to support the idea that the acb1 protein is important for maintaining membrane fluidity at lower and optimal, but not high temperatures. Overall, the authors conclude that acb1 has a key role in membrane remodeling (homoviscous adaptation) at lower and optimal temperatures, but is dispensable at elevated temperatures.*

*To my knowledge, this is the first study of its kind to consider thermal constraints on biotrophic colonization and infection by a fungal pathogen, which is a major strength. I think the manuscript has the potential to make a significant impact on the field, and get the plant-microbe community thinking about the nature of these interactions beyond optimal growth chamber conditions etc. However, I feel a couple of key experiments need further quantitative support.*

We thank the reviewer for their careful assessment of the work.

*Weaknesses*

*A weakness in the manuscript is a lack of quantitation for the FM4-64 membrane trafficking assay (Figure 2C) and the plant infection assays (Fig 2 and 3) - both of which should be relatively easy to address, I hope. I also think the anisotropy assay, given its key importance to the manuscript, needs further explanation in the methods, and possibly some further controls (as outlined in Part II). It would also be compelling to see the fatty acid abundance of a genetically complemented acb1 KO strain return to wild type- like composition, and some of the other important phenotypes (e.g. multiple BIC's) rescued by complementation.*

We thank the reviewer for these excellent suggestions, which, as indicated in detail below, we have taken seriously. To briefly summarize, we have quantified FM4-64 membrane trafficking by focusing on the FM4-64 distribution at the hyphal tip, and we have quantified the plant infection assay. We have provided more information on the anisotropy assay and included more controls. We have repeated all the FAME analyses and most other experiments to include a Δ*acb1 ACB1* complementation strain that rescues the Δ*acb1* phenotype. Unfortunately, due to a dearth of selectable markers and the time frame involved, we did not make the Δ*acb1 ACB1 PWL2-mCherry:NLS BAS4-GFP* complementation strain required for showing rescue of the multi-BIC phenotype by complementation.

***Part II – Major Issues: Key Experiments Required for Acceptance*** *Please use this section to detail the key new experiments or modifications of existing experiments that should be absolutely required to validate study conclusions.

Generally, there should be no more than 3 such required experiments or major modifications for a "Major Revision" recommendation. If more than 3 experiments are necessary to validate the study conclusions, then you are encouraged to recommend "Reject".*

*Reviewer #1: The FAME analysis performed in Figure 1A, B should also be performed on the complemented strain.*

Thank you, we were remiss to not include the complementation strain in the original version of our paper, and we have now repeated all our FAME analyses to include WT, Δ*acb1* and the Δ*acb1 ACB1* complementation strain at all tested temperatures.

*In Figure 3 the authors show that switching to 29°C restores pathogenicity in the mutant. It has been shown that increasing temperature of rice can disrupt the function of some NLR’s. Can this be discounted? Constant incubation at the higher temperatures leading to no infection for all strains could be due to stress of strains. Perhaps in the supplementary data the authors could include another non related mutant that has a similar reduction in pathogenicity at 26°C, and shift it to 29°C to check it’s pathogenicity isn’t restored? I would like to see that this phenomenon the authors report is specific to this mutant only.*

Great point! To address this, we performed the temperature shift experiment (new Fig. 4F,G) using an un-related knockout strain, Δ*ndk1*, encoding the nucleoside disphosphate kinase responsible for modulating intracellular nucleotide pools for redox balance (Rocha and Wilson 2020). In rice cells, Δ*ndk1* is unable to undergo biotrophic growth because it cannot suppress the host oxidative burst, leading to host innate immunity induction (Rocha and Wilson, 2020). However, in the previous study, Δ*ndk1* biotrophic growth was restored when infected cells are treated with DPI to inhibit the host oxidative burst. Thus, the biotrophic growth of this pathogen is dependent on host innate immunity induction. Here, we show that both Δ*ndk1* and Δ*acb1* are nonpathogenic at 26°C at 5 days post inoculation (dpi), but only Δ*acb1* virulence is remediated to levels like that of the WT and complementation strain following a switch to 29 ^o^C. Thus, the phenomenon of temperature-dependent pathogenicity is specific to Δ*acb1* and does not involve, for example, disrupted NLR function that would affect the pathogenicity of unrelated biotrophic mutant strains like Δ*ndk1*. We considered this result was so important we have included it in the main figure.

*What happens to the second acetylCoA gene expression at higher temperatures in the absence of ACB1, does it compensate in any way? The authors suggest it doesn’t because the lipid composition still remains perturbed at 29°C, however, with a perturbed lipid composition at 29°C the mutant can now cause disease. Is that because 29°C increases membrane fluidity in the mutant? For me this wasn’t clear, and I think generation of the double mutant would help clarify this point.*

We thank the reviewer for this comment and the opportunity to correct our paper. We apologize, our choice of words when hypothesizing about another gene with *ACB1*-like function was misleading and has been removed. We originally stated: “We reasoned that increased growth and infection of Δ*acb1* at 29 ^o^C compared to 26 ^o^C resulted from remediation of lipid homeostasis at the elevated temperature, *potentially due to the activation of a second gene with ACB1-like function*”. However, there are no other Acyl-CoA binding proteins encoded in the *Magnaporthe* genome, and here we were musing rhetorically. Therefore, we have removed mention of any putative second Acyl-CoA binding proteins from the text and stress that there is only a single copy of *ACB1* in the genome (which we have re-checked by additional BLAST searches at NCBI), and there are no other proteins with acyl-coA binding domains. Consequently, we could not generate the requested double mutant. Moreover, the remediation of Δ*acb1* at 29 ^o^C is thus due to the tolerance of the changes resulting from the loss of *ACB1* at this temperature rather than remediation of those defects by other means.

*What happens to ACB1 expression at higher temperatures? Is still expressed and temperature overrides its requirement?*

Good question! We have measured *ACB1* expression at 22 ^o^C – 29 ^o^C and find that expression is constitutive (S5 Fig), which is consistent with *ACB1* being required to prevent perturbed fatty acids levels in mycelia at 29 ^o^C.

*The authors claim that loss of ACB1 raised the level of unsaturated fatty acids compared to wild type. An increase in unsaturated fatty acids is generally associated with the membrane being more fluid, but the authors suggest loss of ACB1 leads to a lower membrane fluidity at lower temperatures compared to wild type (based on later anisotropy experiments)? Unsaturated fatty acids have one or more double bonds in their hydrocarbon chains, causing kinks or bends. These kinks prevent the fatty acid chains from packing closely together, which increases the fluidity of the membrane. Can the authors comment?*

Thank you for this comment. As stated above, we repeated the FAME analysis of WT and Δ*acb1* and this time included the Δ*acb1 ACB1* control. Although changes to saturated and saturated fatty acids were observed in Δ*acb1* compared to the other two strains (Fig 1A, *right*), now that we are doing a three-way comparison rather than a two-way comparison, the differences in the saturated:unsaturated ration for WT and Δ*acb1* are no longer significant, therefore we no longer mention this ratio. However, we do now note in the Discussion that membrane fluidity is a complex phenomenon that is influenced by factors other than double bonds in fatty acids, such as the charge and size of head groups, chain lengths and nonglycerol lipid components (sterols and proteins), as described by Cassim et al. (2019). Understanding these changes in *M. oryzae*, which have not been examined before, is beyond the scope of the current paper, but warrants investigation in the future.

*The authors show that appressoria can normally function, in that they can generate enough pressure to penetrate the host leaf cuticle. Does that mean the role of ACB1 in lipid homeostasis is specific to invasive hyphal growth/BIC formation? I would have thought the alterations in membrane composition would also influence appressorium function? Cao et al., 2023 also characterised this gene and suggested the mutant displayed a reduction in appressorium specific turgor pressure.*

Great suggestion! We measured turgor as suggested and find, like Cao et al., 2023, that Δ*acb1* appressorial turgor is reduced compared to WT at 26 ^o^C (S1A Fig), although penetration on host leaves still occurs with enough frequency to cause disease. Interestingly, at 29 ^o^C, all strains were reduced for turgor compared to WT at 26 ^o^C (S1B Fig). This may underlie the findings that WT does not penetrate leaf cuticles at 29 ^o^C. In summary, your hypothesis was correct, and we now provided supporting experimental evidence.

*What happens if ACB1 is overexpressed? Is this strain more pathogenic at lower temperatures and compromised at higher temperatures?*

This was an interesting suggestion. We had a Δ*acb1* complementation strain expressing *ACB1* under the constitutive *RP27* promoter, which we now include here. S6 and S7 Figs show that this strain is not altered in radial growth or infection compared to WT and the Δ*acb1 ACB1* (ie under native promoter) strains, even though there are some small changes to fatty acid abundances compared to WT. Therefore, the strain is not more pathogenic at lower temperatures.

*Line 153 the authors say ‘In contrast, dacb1 IH accumulated…’ however it is vegetative hyphae displayed in Figure 2C? The authors suggest in line 151 that the loss of ACB1 did not effect endocytosis. However, have the authors considered what other proteins may be effected in function at low and optimal growth conditions in the mutant? For example, other membrane associated proteins like flippases? In fact, it has been reported that FM4-64 internalisation can be taken up via two pathways, by endocytosis or by internalisation involving flippase and lipid transfer protein activity, which can lead to different patterns of staining. Although endocytosis may remain unaffected, membrane bound proteins such as flippases may effected in the mutant?*

Thank you for catching this mistake, we have now changed this to read “In contrast, Δ*acb1* vegetative hyphae..” The suggestions about flippases and the other pathways mediating FM4-64 uptake are interesting and valid. However, examining flippases directly was beyond the scope of this work. Thus, we have added the following caveat to the results section: “..although we cannot entirely rule out that other FM4-64 uptake pathways such as those mediated by membrane-bound flippases are not unaffected..”

*The images in Figure 2C are not very convincing for me, and I feel the conclusion being drawn, that ACB1 has a temperature dependent role in membrane trafficking is a strong conclusion based on the images/data provided. Do GFP tagged membrane trafficking proteins, for instance, display a change in their localisation pattern in the mutant at 22 and 26 compared to WT to verify what is being observed with the FM4-64 stain? These images need quantifying. It also seems like z stack projections have either not been provided, or the tips of hyphae have not stained well, or in the mutant strain the tips of hyphae are aberrant- for example is the spitzenkorper disrupted?*

We deeply appreciate this comment. For time considerations, we did not look at GFP-tagged membrane proteins, however we did include Δ*acb1 ACB1* complementation strain for a better comparison of membrane staining across the strains. We provide merged z-stack projections and although we cannot comment on the spitzenkorper directly, we have now quantified the lack of tip staining in Δ*acb1* (but not the other strains) to show it is significantly reduced at 22 – 26 ^o^C but remediated at 29 ^o^C (new Fig 3D). We believe this is an important contribution to the work and we thank the review for the suggestion.

*The authors show by DPH fluorescence anisotropy that the WT at 22-32 maintains membrane fluidity homeostasis. However, FAME experiments show that the WT fatty acid composition changes as you incubate 22-29. Statistical analysis needs to be performed between wild type samples as temperature changes for the FAME experiment in S3. Are these two results contradicting? Altered fatty acid composition effects the levels of saturated vs unsaturated fatty acids within the membrane which in turn effects the membrane fluidity? Please could the authors comment?*

When we looked at individual fatty acids in WT, we found they did not change in abundance in response to temperature. As noted above, the findings from the FAME analysis and membrane fluidity data are not contradicting because other components likely affect membrane fluidity, and we have now added a few sentences to the Discussion section to address this.

*In order to carry out the staining cells were permeabilised with Amphotericin B, can the authors to sure that the increased fluorescence observed in the mutant isn’t due to other effects knocking out the gene may have on the cell wall for example, which could lead to enhanced permeabilization and subsequent uptake of dye in the mutant vs WT?*

We thank the reviewer for this comment. Here, we have repeated the anisotropy assay without the use of Amphotericin B, which we found was not necessary for the measurements. Thus, Amphotericin B has been removed from the text and does not account for any enhanced permeabilization and dye uptake in the mutant strain. However, values were similar whether or not Amphotericin B was used. Anisotropy is a ratio determined from the rotational values of DPH in the membrane, and is independent of DPH concentration. Thus, this assay is agnostic of any cell wall integrity differences between the different strains, as any DPH trapped in the cell wall (for example) would not rotate. A sentence was added to the Results section to stress this point.

*Why do the authors not provide images of the samples that were used to generate the graph?
I would like to see all the data points and replicates on the graph. N=3 how did the authors take measurements, did they take an average of multiple technical reps and then repeat this 3 times? The error bars are also very large. For me this is the weakest part of the manuscript and this experiment needs revising.*

Thank you for your comments. We did not provide images of the samples because none were taken. Stained hyphae were transferred to a cuvette and measured for fluorescence signal in a spectrophotometer. Thus, only quantitative data was generated in this assay. Here, we have improved the visualization of the data by switching to boxplots shown side-by-side for each temperature, which should provide more information about the variance of the data. You are correct, four technical replicates were done for each of the three biological replicates. We recorded absorbance from two polarized planes to assess mobility of the probe (DPH) in our sample. Means were corrected by subtracting background signal, as determined by non-DPH stained samples. Increasing the number of technical reps and ensuring equal cell concentration decreased the size of the error bars. We also included a control showing how DPH measures membrane fluidity by measuring fluorescence at constant temperature but in the presence of increasing concentrations of DMSO, which destabilizes and permeabilizes the cell membrane (S8 Fig). Our new ion leakage data (Fig 6B) also corroborates the DPH results.

*Reviewer #2: (No Response)*

*Reviewer #3: Fig 2. I don't think the data presented in this figure is sufficient to support the conclusion that membrane trafficking is altered in acb1 mutants. I think the FM4-64 assay in principle is okay, but the authors need to find a way to make this quantitative and more robust, rather than showing single representative images. It would also be more compelling to include data to show that the complementation strain is restored to wildtype-like behavior in this assay.*

We thank the reviewer for their constructive remarks. In addressing this, as suggested, we have now included images and data from the complementation strain, and quantified the rates of FM4-64 staining at hyphal tips (depleted in Δ*acb1* at 22 – 26 ^o^C but restored in Δ*acb1* at 29 ^o^C) (new Fig 3C,D). Considering FM4-64 is used to investigate endocytosis and vesicle trafficking in living eukaryotic cells, we are now more confident that we are observing defects in membrane trafficking in the Δ*acb1* strain compared to WT, as predicted from yeast studies.

*I also think the authors should make some effort to quantify disease symptoms on leaves to increase the robustness of these findings. For example quantifying lesion size etc.*

Great suggestion! We have now included graphs for all plant infection data showing lesion coverage on whole leaves with three technical and biological replicates. This quantitative data corroborates our findings from these images.

*Fig 4. I would like to see the individual data points plotted for this graph, and a more complete description of the assay in the methods. It's not clear what a replicate is in this experiment, or precisely how the measurements were taken. Is this assay done in cuvettes using mycelia? Are the mycelium homogenized etc? I can see how this assay would work for single-celled yeast, but it's less obvious how it would work with filamentous fungi. I'd also like to see more biological replicates if possible - the error bars for the mutant, especially at the higher temperature are quite large. The conclusion that membrane fluidity in acb1 mutants at high temperatures is not significantly different from WT at 29 and 32 degrees, needs strengthening. Ideally, and perhaps this is asking too much, I'd like to see further evidence that this assay can reliably detect changes in membrane fluidity in control hyphae - perhaps in response to drugs known to perturb membrane properties, as in the cited studies. I'm hoping that this will be relatively straightforward for the authors to do, and will make these important findings more robust.*

We thank the reviewer for these excellent and important comments. We have repeated and revised the original Fig 4 graph (now Fig 6A). We no longer use Amphotericin B and several other refinements to the protocol have led to a reduction in error bars (the data is now presented as a box plot). We have included a better description of the method, which should clarify how many replicates were performed and how the measurements were taken. Briefly, we repeated the experiment with more technical replicates and ensured equal cell concentration of fungal cells in cuvettes with OD_600_ which reduced the size of our error bars. The method section was updated accordingly. Cultures were grown in liquid media and not homogenized in this experiment. We were inspired by the methodology of Zhang *et al.* (2013) where *Rhizoctonia solani*, another filamentous fungi, was successfully stained with DPH without the need of homogenization. For the requested positive control, we added dimethyl sulfoixde (DMSO), which binds within the cell membrane and increases membrane fluidity (Kundu et al. 2021), to mycelia and found that 0.5% DMSO drastically lowers anisotropy, while concentrations of 1% DMSO and above set anisotropy to zero, demonstrating that anisotropy values in our data indeed serve as proxies for membrane fluidity. The graph showing the effect of DMSO on anisotropy is included in our supplementary data (S8 Fig). We also now incorporate an ion leakage assay (Fig 6B) which corroborates and strengthens the membrane fluidity findings.

***Part III – Minor Issues: Editorial and Data Presentation Modifications***
*Please use this section for editorial suggestions as well as relatively minor modifications of existing data that would enhance clarity.*

*Reviewer #1: Figure 1D the multiple BICs highlighted in the red boxes need quantifying. I think it would be good to have a zoomed in micrograph of the wild type BIC phenotype for comparison for readers who are less familiar with this topic.*

Good suggestions. We included zoomed-in images of BIC phenotypes from WT and mutant. We have also quantified the number of BICs/ BIC fragments (new Fig. 2C)

*To complement the FAME analysis in Figure 1A, B have the authors considered to quantify/visualise changes in the plasma membrane ridgity and composition of vegetative hyphae and appressoria using the probes N+-BDP (for tension changes) and NR12S (chemical polarity and lipid phase) published by Michels et al., 2021?*

Good suggestions! Firstly, while we did not use the suggested probes, we did incorporate another mycelial assay quantifying ion leakage, which increases in Δ*acb1* at 22-26 ^o^C but not 29 ^o^C compared to WT, indicating that plasma membrane integrity is affected in Δ*acb1* in a manner consistent with the membrane fluidity results. This new data corroborates the membrane fluidity data.

Secondly, as pointed out earlier, the FAME analysis results do not directly translate to changes in membrane properties, given that other factors are involved as well.

Thirdly, although we felt that using these specific probes was beyond the scope of the current paper given our lack of expertise in this area, the new appressorial turgor data, showing impaired turgor in all strains at 29 ^o^C, warrants their future use. We have written a paragraph in the Discussion about these probes and their utility to future work on temperature-dependent appressoria function.

*Have the authors considered making a model summarizing their proposed function of ACB1?*

Unfortunately, we have not provided a model because it is currently unknown in any system how Acb1 functions to transport Acyl-CoAs to different downstream enzymes to maintain lipid homeostasis. Thus, we did not want to speculate at this time.

*Reviewer #2: Figure 2A: Please enlarge the data points (circles) in the bar graph.*

We enlarged the data points in the bar graph.

*Figure 2A: The growth assay illustration is extremely convincing. The authors could also show a graph showing the three replicates of the growth assay.*

Thank you, this is now included in S2A Fig.

*Figure 3A and C: The virulence assay data is very convincing. However, if the authors could show the quantification, such as number of lesions/leaf area or diseased area, it would make the data even more convincing.*

Thank you for this comment. We have quantified lesion coverage on whole leaves throughout.

*Reviewer #3: I'm a little curious as to why WT replicates were omitted from the FAME analysis at 26 and 29 degrees.*

We repeated all FAME analyses to include the Δ*acb1 ACB1* complementation strain and all treatments now have three biological replicates. Previously, two replicates were omitted because they failed during the FAME analysis.
